# Preparation for mice spaceflight: Indications for training C57BL/6J mice to adapt to microgravity effect with three-dimensional clinostat on the ground

**DOI:** 10.1016/j.heliyon.2023.e19355

**Published:** 2023-08-24

**Authors:** Chenchen Song, Taisheng Kang, Kai Gao, Xudong Shi, Meng Zhang, Lianlian Zhao, Li Zhou, Jianguo Guo

**Affiliations:** Key Laboratory of Human Disease Comparative Medicine, Chinese Ministry of Health, Key Laboratory for Animal Models of Emerging and Reemerging Infectious Diseases, Institute of Laboratory Animal Science, Chinese Academy of Medical Sciences and Comparative Medicine Center, Peking Union Medical College, Beijing, China

**Keywords:** Microgravity, Three-dimensional clinostat (3D clinostat), Random positioning machine (RPM), Bone loss, Serum metabolomics, Animal model

## Abstract

Like astronauts, animals need to undergo training and screening before entering space. At present, pre-launch training for mice mainly focuses on adaptation to habitat system. Training for the weightless environment of space in mice has not received much attention. Three-dimensional (3D) clinostat is a method to simulate the effects of microgravity on Earth. However, few studies have used a 3D clinostat apparatus to simulate the effects of microgravity on animal models. Therefore, we conducted a study to evaluate the feasibility and effects of long-term treatment with three-dimensional clinostat in C57BL/6 J mice. Thirty 8-week-old male C57BL/6 J mice were randomly assigned to three groups: mice in individually ventilated cages (MC group, n = 6), mice in survival boxes (SB group, n = 12), and mice in survival boxes receiving 3D clinostat treatment (CS group, n = 12). The mice showed good tolerance after 12 weeks of alternate day training. To evaluate the biological effects of simulated microgravity, the changes in serum metabolites were monitored using untargeted metabolomics, whereas bone loss was assessed using microcomputed tomography of the left femur. Compared with the metabolome of the SB group, the metabolome of the CS group showed significant differences during the first three weeks and the last three weeks. The KEGG pathways in the late stages were mainly related to the nervous system, indicating the influence of long-term microgravity on the central nervous system. Besides, a marked reduction in the trabecular number (*P* < 0.05) and an increasing trend of trabecular spacing (*P* < 0.1) were observed to occur in a time-dependent manner in the CS group compared with the SB group. These results showed that mice tolerated well in a 3D clinostat and may provide a new strategy in pre-launch training for mice and conducting relevant ground-based modeling experiments.

## 1 Introduction

The complex environment of space, including microgravity, radiation, changes in circadian rhythm, and extreme temperatures, can impact several biological processes [[1], [2], [3], [4], [5], [6]]. Microgravity is one of the critical problems that astronauts face in space. Many studies [[7], [8], [9], [10], [11], [12]] have shown that long-term weightlessness can lead to various physiological and pathological changes, including in the immune, nervous, reproductive, and cardiovascular systems and in skeletal muscle tissues. To adapt to the weightless environment of space, astronauts need to go through a series of rigorous training before going into space, such as parabolic aircraft flights, immersion and head down tilt. Mice are the most commonly used experimental animals in space for their small size and thus increasing space utilization and the cost-efficiency ratio. Many space experiments using mice have been conducted aboard the Space Shuttles, such as ‘BION’, an unmanned biosatellite, and the International Space Station (ISS). Launched in 2013, the Russian Bion-M1 biosatellite accommodated 45 male mice in groups for 30 days [13]. Launched in 2009, 6 male mice housed individually in the Italian Mice Drawer System (MDS) were exposed for 91 days, the longest duration rodent experiment ever conducted in space [14]. Unfortunately, in the two Long-term experiments on mice in space, more than half of the animals died because of hardware malfunctions or unpredictable reasons. Although, with the improvement of habitat hardware, the survival rate of mice in space was improved, the effects of space special environment such as microgravity on the survival rate and survival state of mice should not be ignored [15]. During space launch, astronauts and experimental animals experienced a sharp change in gravity, from the normal 1 g Earth gravity to the 3–8 g hypergravity during launch, and the 10^−4^-10^−3^ g microgravity after entering space. The rapid change of gravity and the unprecedented space microgravity environment would cause acute stress injury to experimental animals. The good condition of spaceflight mice was the basis for the positive results of space animal experiments. Like astronauts, animals need to undergo training and screening before entering space. However, pre-launch training for mice at present mainly focuses on adaptation to habitat system. Training for the weightless environment of space in mice has not received much attention.

Currently, weightlessness studies are mainly selected by relying on animal models [16,17], and the rodent hindlimb unloading model is the most widely used [18,19] to study spatial effect. However, this model has certain limitations in that it applied locally and not to the whole animal [20]. Clinostats have invited the attention of researchers because experiments involving microgravity in space are expensive and conducted infrequently, and the time frame for producing a microgravity environment on ground is generally too short to meet the requirements of most biological experiments. A previous study has shown that the three-dimensional (3D) clinostat is a good method to simulate the effect of microgravity on ground, and the tested object experiences zero total force in the entire experimental process through random clinostat [21]. The clinostat is not like a drop tower that only provides a microgravity environment but it can also be used as an effect of microgravity simulation equipment [21]. Previous studies [[22], [23], [24], [25], [26], [27], [28], [29]] have used the 3D clinostat to simulate the effect of microgravity on plants, cells, and *Caenorhabditis elegans* but not on mice. Compared with the classical tail suspension model, the 3D clinostat model could produce a whole-body microgravity simulation effect, and may better simulate the vestibular signal disorder as well as the resulting perception conflict and central system abnormalities. Besides, unlike humans, the microgravity space environment would have less significant effect on cerebral blood flow in mice as a four-limb landed animal. For microgravity environment adaptability training, 3D gyration model may be more suitable than tail suspension model. Therefore, we conducted a study to evaluate the feasibility and effects of long-term treatment with three-dimensional clinostat in C57BL/J mice, trying to establish a microgravity environment adaptation method and evaluate simulated microgravity effects in mice.

## 2 Methods

### 2.1 Structure of the 3D clinostat

**Fig. 1:**
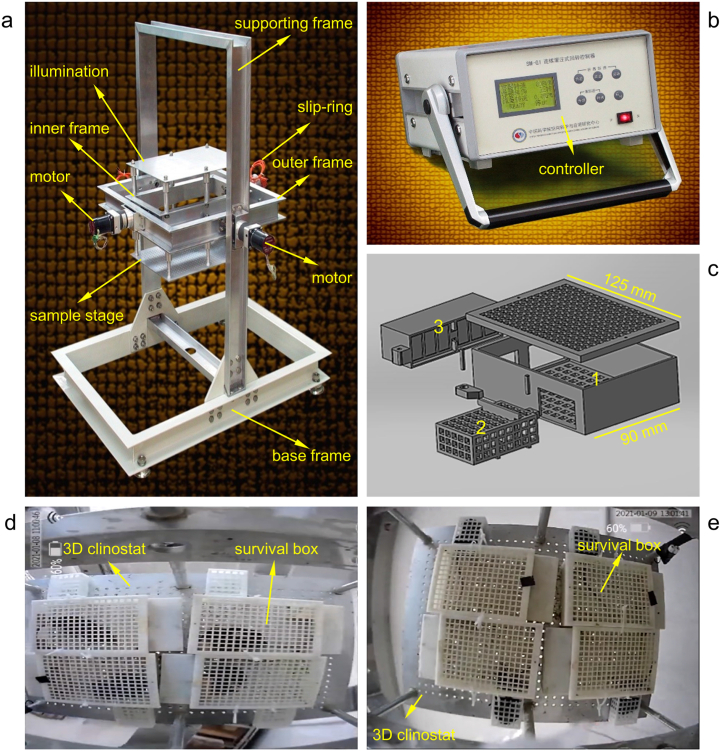
(a) Three-dimensional clinostat and (b) the controller. (c) Mouse survival box (SB) pattern; 1: Activity area. 2: Rest area. 3: Food and water areas; (d) Adaptive training of the clinostat (CS) group on the 3D clinostat; (e) Mice in the CS group on the 3D clinostat during the study.

The Chinese Academy of Sciences, Beijing, China, manufactured a 3D clinostat apparatus in accordance with the original design by Hoson [30], and its operation was controlled via a rotation control system (Fig. 1a and b).

The clinostat is comprised of a base frame, a supporting frame, an outer frame, and an inter frame (Fig. 1a). This is similar to the 3D clinostat developed by Hoson [30]. The sample stage and illumination are placed on both sides of the inter-frame in parallel, and they are 170 mm away from the shaft. The inner frame is fixed on the outer frame, which rotates about the horizontal axis with its rotating axes perpendicular to each other. Additionally, the rotational motion of the two rotating frames is driven by stepper motors. A controller with a single-chip microcomputer regulates the motor rotational speed, direction, and running duration. A slip ring is used to perform all incoming and outgoing electrical wiring. Moreover, the rotating frames are the outer frame and inner frame. It can rotate independently and can be used to simulate the microgravity effect of larger plants, seeds, and microorganisms. Specific parameters: Loading platform: area, 400 mm × 300 mm; distance from the center, 170 mm; bearing capacity, 0–3 kg; and illumination, 400 mm × 300 mm. The sample stage can hold up to 12 sample containers with a diameter of 90 mm. Opposite the sample stage distance from the center point, 170 mm, removable. The lighting power is adjustable in the range of 0–50 w, and the light source is a white LED divided into two groups. The light is adjustable (5 cm under the light plate, 80–800 μmol/m^2^/s). The rotation modes are as follows: random rotation speed, 0–10 rpm; speed resolution, 0.1 rpm. The overall size of the 3D clinostat is 1000 mm × 912 mm × 1250 mm, and the total weight is 35 kg.

## 3 Mice and treatments

The Ethics Committee of the Laboratory of Animal Science of Peking Union Medical College approved all experimental procedures. The Animal Ethics is IACUC-20220415. Animal care was provided in accordance with institutional guidelines and all animal studies were performed in compliance with the ARRIVE guidelines. 8-Week-old male C57BL/6 J mice were purchased from Beijing HFK Bioscience Co., Ltd and kept in the experimental room at temperature 22 ± 2 °C, humidity 40 ± 5%, under a 12-h light/dark cycle. The mice were randomly divided into three groups: mice in individually ventilated cages (MC group, n = 6), mice in survival boxes (SB group, n = 12), and mice in survival boxes receiving 3D clinostat treatment (CS group, n = 12). As the 3D clinostat rotates 360°, we took advantage of the burrowing habits of the mouse and designed a survival box to keep the mice fixed on the 3D clinostat (Fig. 1c). From Fig. 1c, represents the free movement area of the mouse, 2 represents the rest area of the mouse, 3 represents the food and water supply area. In the third area, we put feed and solid AGAR to provide water. Only one mouse was placed in one survival box; therefore, fighting and biting among mice were avoided.

The activity of the mice makes it impossible for it to be uniform, and the total force cannot be zero. However, binding or other ways to limit movement will result in stress injury to the mouse. When using a clinostat to simulate microgravity, the mice could drill into the survival box and stay in the rest area. It can be used as a mass point to ensure that the total force applied to the experimental animal is near zero and to perform simulated microgravity experiments in 3D clinostat safely and stably (Fig. 1d and e).

Before the study, we conducted adaptive training for the mice in the CS group to relieve stress. The adaptive training process involved the following: the mice in the CS group were placed on the 3D clinostat, and we provided standard pellet food and 2% agar gel (water supply) in the survival boxes. The CS group underwent adaptive training for 5 days and rotated for 1 h, 2 h, 4 h, 8 h, and 12 h each day and rested for 2 days before starting the study. Meanwhile, the MC group was always placed in IVCs and the SB group was always placed in survival boxes without any treatment. Water and food were freely available, and all mice were observed daily.

### 3.1 Simulated microgravity

8-week-old mice were acclimated to the facility environment for 2 days and underwent adaptive training to 3D clinostat for 5 days. The formal experiments began at 9 weeks old. The CS group experienced simulated microgravity on clinostat for 1 day and rested for 1 day; the SB group was placed in the survival boxes in according with CS operation frequency; the MC group was always placed in the individually ventilated cages. The experiment lasted for 12 weeks. Left femur from the CS and/or SB groups were collected weekly. Serum and left femur from the MC group were collected every 2 weeks. The left femur was wrapped in saline-soaked gauze and stored at −20 °C for *ex vivo* microcomputed tomography analysis, and the serum was stored at −20 °C for metabolomics. The mice were anesthetized by intraperitoneal injection of pentobarbital sodium (50 mg/kg) and blood samples were collected from inferior vena cava. After sample collection, the mice were sacrificed by cervical vertebra twisting.

### 3.2 Micro-CT imaging of bone

CT imaging was conducted using a Siemens INVEON scanner with the following settings: tube voltage, 60 kV; tube current, 400 μA; and exposure time, 800 m s over 360° rotation. The Feldkamp filtered back-projection algorithm was used to reconstruct the images. Images acquired from the scanner were viewed and analyzed using INVEON Workplace software.

## 4 Extraction of metabolites

To extract metabolites from serum samples, 400 μl of cold extraction solvent methanol/acetonitrile/H_2_O (2:2:1, v/v/v) was added to 100 μl of serum sample and adequately vortexed. After vortexing, the samples were incubated on ice for 20 min and then centrifuged at 14,000 *g* for 20 min at 4 °C. Finally, the samples were redissolved in 100 μl of acetonitrile/water (1:1, v/v) solvent for liquid chromatography–mass spectrometry (LC–MS) analysis and transferred to LC vials.

### 4.1 LC–MS analysis

For the untargeted metabolomics of polar metabolites, extracts were analyzed using a quadrupole time-of-flight mass spectrometer (Sciex Triple TOF 6600) coupled to hydrophilic interaction chromatography via electrospray ionization by the Shanghai Applied Protein Technology Co. Ltd. The LC separation was on an ACQUIY UPLC BEH Amide column (2.1 × 100 mm, 1.7-μm particle size) (Waters, Ireland) using a gradient of solvent A (25-mM ammonium acetate and 25 mM ammonium hydroxide in water) and solvent B (acetonitrile). The gradient was 85% B for 1 min and was linearly reduced to 65% in 11 min, and then was reduced to 40% in 0.1 min and maintained for 4 min, and then increased to 85% in 0.1 min, with a 5-min re-equilibration period. The flow rate was 0.4 mL/min, the column temperature was 25 °C, the autosampler temperature was 5 °C, and the injection volume was 2 μl. The mass spectrometer was operated in both negative and positive ionization modes. The ESI source conditions were set as follows: ion source gas 1 (gas 1) as 60, ion source gas 2 (gas 2) as 60, curtain gas (CUR) as 30, source temperature: 600 °C, IonSpray Voltage Floating (ISVF) ± 5500 V. In auto MS/MS acquisition, the instrument was set to acquire over the *m*/*z* range 25 to 1000 Da, and the accumulation time for production scan was set at 0.005 s/spectra. The production scan was acquired using information-dependent acquisition with selected high sensitivity mode. The parameters were set as follows: the collision energy was fixed at 35 V with ±15 eV; declustering potential 60 V (+) and −60 V (−); exclude isotopes within 4 Da; candidate ions to monitor per cycle: 10.

### 4.2 Data analysis

The raw MS data (wiff. Scan files) were converted to MzXML files using ProteoWizard MS Convert before importing into freely available XCMS software. For peak picking, the following parameters were used: centWave *m*/*z* = 25 ppm, peak width = c (10, 60), prefilter = c (10,100). For peak grouping, bw = 5, mzwid = 0.025, and minfrac = 0.5 were used. Only the variables with more than 50% of the nonzero measurement values in the extracted ion features were kept in at least one group. MS/MS spectra identified metabolite compounds with an in-house database established with available authentic standards. After normalization to total peak intensity, the processed data were uploaded before importing into SIMCA-P (version 14.1, Umetrics, Umea, Sweden). They were subjected to multivariate data analysis, including Pareto-scaled Principal component analysis (PCA) and orthogonal partial least-squares discriminant analysis (OPLS-DA). Sevenfold cross-validation and response permutation testing were used to evaluate the robustness of the model.

### 4.3 Analysis of bioinformatics

For KEGG annotation of pathways, the metabolites were blasted against the online KEGG database to retrieve their COs and were subsequently mapped to pathways in KEGG.

### 4.4 Statistical analysis

The data are shown as means ± SEM. GraphPad Prism 9.0 software was used for statistical analysis. Two-way ANOVA followed by post-hoc test evaluated the significance of differences in the mouse body weight and bone parameters between groups. A difference was considered significant if the *P*-value was <0.05. Nonmetric multidimensional scaling (NMDS) was conducted based on the Bray–Curtis distance. The corresponding KEGG pathway enrichment analyses were applied based on Fisher's exact test, considering the total metabolites of each pathway as a background dataset. Only pathways with *P*-values <0.05 were considered significantly changed pathways.

## 5 Results

### 5.1 Effects of 3D clinostat on the body weight and general status in mice

**Fig. 2:**
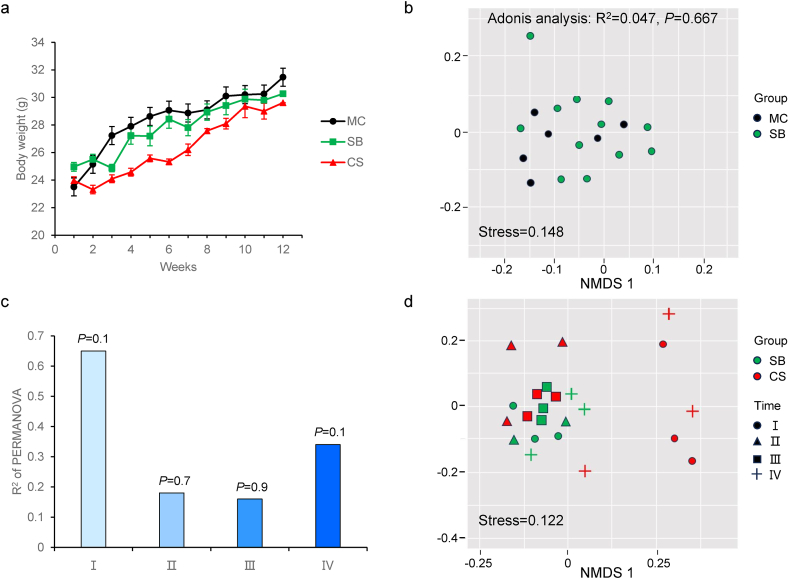
Effects of 3D clinostat on body weight and bone parameters in mice. (a) Weekly body weight chart of the mice. (b) NMDS analysis of metabolic pattern in mouse cage (MC) and survival box (SB) group. (c) R^2^ of permanova between 3D clinostat (CS) group and SB group mice metabolic patterns in different time regions. (d) NMDS analysis of differences characteristics of CS and SB group mice metabolic patterns in different time regions. Ⅰ: 1–3 weeks, Ⅱ: 4–6 weeks, Ⅲ: 7–9 weeks, Ⅳ: 10–12 weeks. *P < 0.05 (CS versus MC), #*P* < 0.00.05 (CS versus SB), $ *P* < 0.05 (SB versus MC).

A veterinarian having expertise in the medical care of mice evaluated the animals daily during the study. Through the course of the study mice were clinically normal, with no physical or behavioral abnormalities noted. The gain in body weight was similar for mice in all experimental groups, further demonstrating the lack of any observable differences in health status (Fig. 2a). Together, these findings suggest that mice adapt well to the 3D Clinostat without adverse health effects.

### 5.2 Effects of 3D clinostat on the metabolic patterns in mice

Studies have shown [[31], [32], [33]] that microgravity changes metabolites in the body, and therefore we examined the metabolomics of serum. Serum specimens were subjected to untargeted metabolomics analysis to identify the hematic metabolic profiles among the groups tested. NMDS analysis showed that there were no significant differences between SB and MC groups (Fig. 2b). It indicated that survival boxes itself did not affect the metabolic pattern of C57BL/6 J mice.

**Fig. 3:**
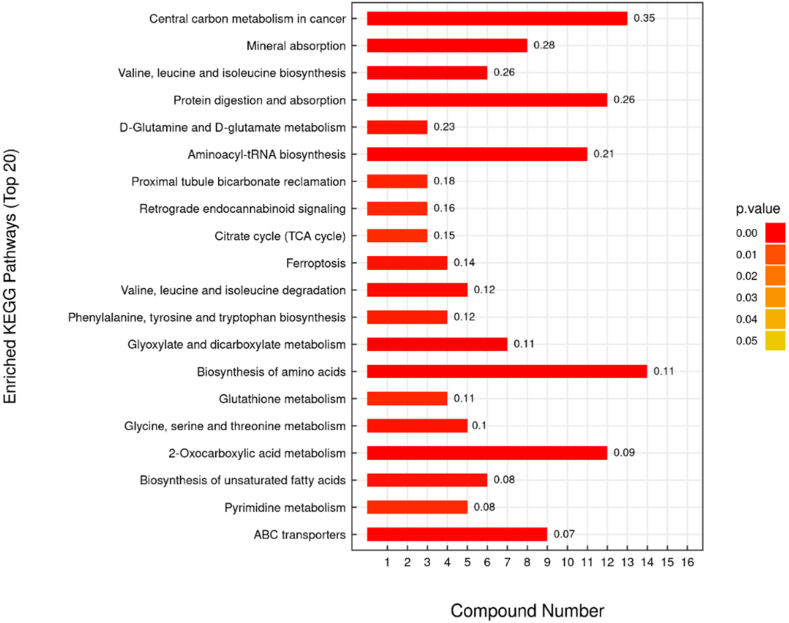
Differential metabolites involved in enrichment analysis of signaling pathways in the stage Ⅰ.

To explore the effect of 3D clinostat, we performed analysis for metabolic patterns in the SB group and CS group in different time regions. NMDS analysis showed that compared with the SB group, stage Ⅰ and Ⅳ have a separation in the CS group (Fig. 2c and d), while stage Ⅱ and Ⅲ did not separate well between the SB and CS groups. Differences between groups were shown in Fig. 2b, our results showed that p value of the stage Ⅰ and Ⅳ was 0.1. Table 1 showed that the differential metabolites changed most significantly in the stage Ⅰ. Meanwhile, compared with the stage Ⅱ, the differential metabolites of the stage Ⅲ and Ⅳ were increasing with time-dependent. It suggested that after 3D clinostat treatment, mice have a stress response in the stage Ⅰ and need to adapt and the number of differential metabolites in mice increased with the time of microgravity simulation. We then analyzed the KEGG pathway, in which the differential metabolites known in the stage Ⅰ were enriched (Fig. 3). As expected, we found metabolic pathways associated with stress responses, including Retrograde endocannabinoid signaling.

**Table 1:** Differential metabolite statistics.

		Ⅰ	Ⅱ	Ⅲ	Ⅳ	Total metabolites
All	Changed	3036 (0.199)	246 (0.016)	365 (0.023)	867 (0.056)	15,226
	Up	1766 (0.115)	148 (0.009)	195 (0.012)	377 (0.024)	2486
	Down	1270 (0.103)	98 (0.006)	170 (0.011)	490 (0.032)	2028
Matched	Changed	233 (0.156)	21 (0.014)	30 (0.020)	56 (0.037)	1492
	Up	150 (0.100)	16 (0.010)	12 (0.008)	35 (0.023)	213
	Down	83 (0.055)	5 (0.003)	18 (0.012)	21 (0.014)	127

Note: The numbers outside the brackets represented the number of differential metabolites; the numbers in parentheses represented the ratio of differential metabolites to total metabolites.

**Fig. 4:**
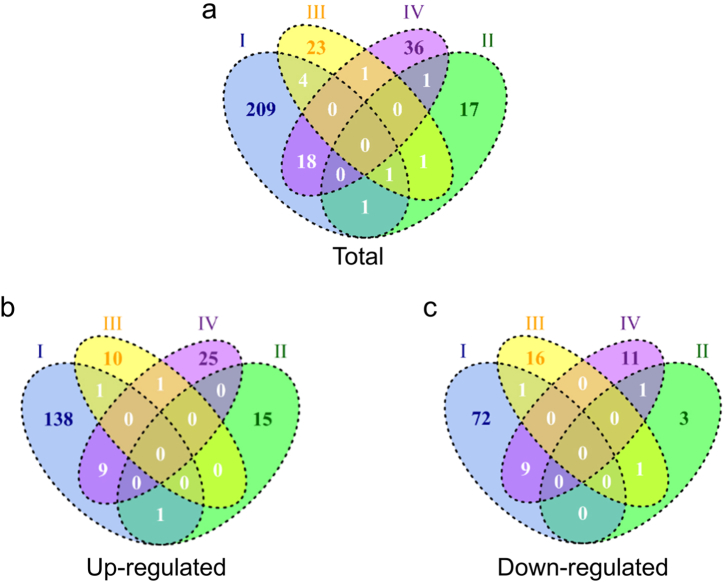
Venn of the differential metabolites. (a) Total differential metabolites (known). (b) Up-regulated differential metabolites (known). (c) Down-regulated differential metabolites (known). Ⅰ: 1–3 weeks, Ⅱ: 4–6 weeks, Ⅲ: 7–9 weeks, Ⅳ: 10–12 weeks.

**Fig. 5:**
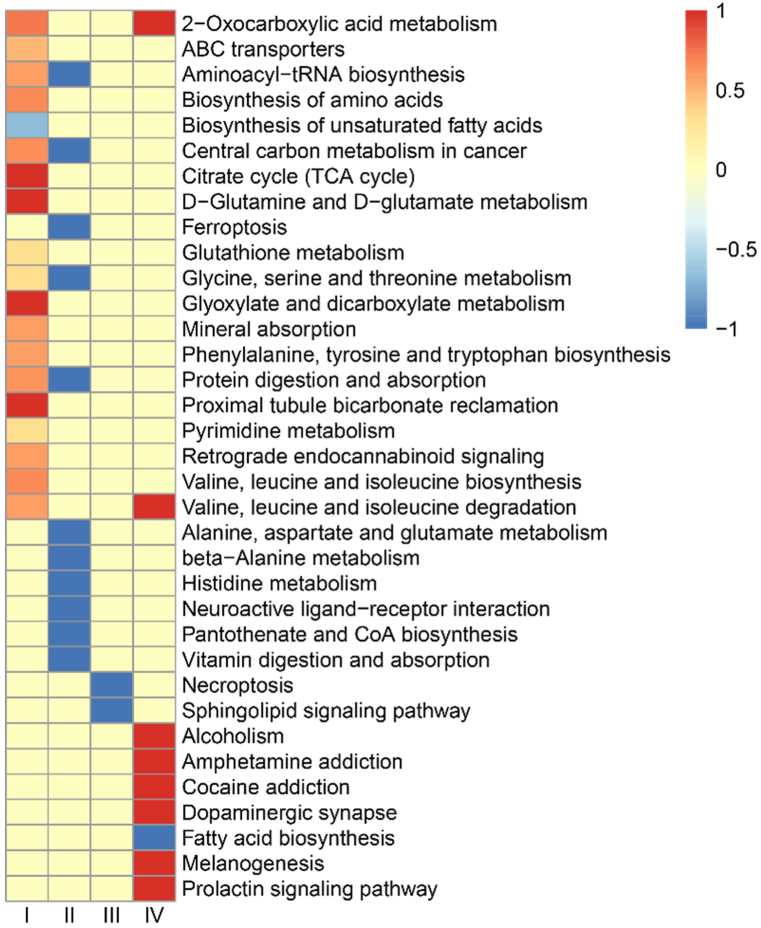
KEGG pathway enriched with differential metabolites at all stages. Ⅰ: 1–3 weeks, Ⅱ: 4–6 weeks, Ⅲ: 7–9 weeks, Ⅳ: 10–12 weeks.

Besides, there are 28 common differential metabolites between the stage Ⅰ and Ⅳ in the Venn diagram (Fig. 4a–c). Table 2 showed that the common differential metabolites mainly focus on the tumor, neurotoxicity and inflammation. Further, we performed pathway analysis in different stage using the KEGG metabolic library. Fig. 5 showed that there were clearly differences in different stages. In stage I, some metabolic related pathways were activated. In stage Ⅱ and Ⅲ, pathways activated in stage I were recovered along with several pathways downregulated. With the prolongation of the 3D clinostat processing time, 8 signaling pathways were observed to be activated in the stage Ⅳ, including 2-Oxocarboxylic acid metabolism, Valine, leucine and isoleucine degradation, Alcoholism, Amphetamine addiction, Cocaine addiction, Dopaminergic synapse, Melanogenesis and Prolactin signaling pathway, which may be related to the change of central nervous system. And pathway related to fatty acid biosynthesis was inhibited in stage IV. Notably, we found that 2-Oxocarboxylic acid metabolism and Valine, leucine and isoleucine degradation overlapped in the stage I and IV. These results suggested that during the early stage I, the mice mainly displayed acute and stress response to the rotation. After that, the mice gradually adapted to the situation during stage Ⅱ and Ⅲ and the acute and stress effects of microgravity were recovered. Further, with the extension of simulated microgravity time, the long-term effects gradually emerged, especially the changes related to the dopamine and central nervous system.

**Table 2:** The common differential metabolites between the stage I and stage IV.

Name	fold change I	fold change IV	Function	Related disease	Reference
Ginsenoside re	2.96	1.64	nerve protection, anti-cancer	cancer, neurodegeneration disease	[34]
Hexadecanedioic acid, 3,3,14,14-tetramethyl-	2.49	0.7	–	–	–
Methylmalonic acid	2.43	1.3	drive tumor aggressiveness	tumor	[35]
6-phospho-d-gluconate	2.01	1.26	glycolysis intermediate	systemic lupus erythematosus	[36]
Kynurenic acid	1.94	4.45	nerve protection	Alzheimer's disease	[37]
L-Tyrosine	1.78	2.08	melanin formation substrate	melanin	[38]
Delsoline	1.57	1.3	gangliolysis	muscle tension and overexertion	[39]
Coproporphyrin i	1.47	0.81	biomarker of OATP1B Activity	Rheumatoid Arthritis	[40]
Cis,*cis*-muconic acid	1.02	0.84	C6 dicarboxylic acid platform chemical	the production of drugs	[41]
Propylene glycol propyl ether	−0.32	−0.23	toxicity	tumors	[42]
Chlorhexidine	−0.5	−0.74	antimicrobial	gingivitis	[43]
Pentadecanoic acid	−0.61	−0.48	promoted glucose uptake	type 2 diabetes	[44]
Norharmane	−0.67	−0.7	protective against neurodegenerative diseases	Alzheimer's disease	[45]
Oxindole	−0.78	−0.79	kinase inhibitors	cancer	[46]
Gln-val	−0.84	−0.89	melanoma cells to metastasize to the liver	melanoma	[47]
2,5-dimethoxy-4-propylphenethylamine	−0.86	−0.79	induce neurotoxicity	neuroinflammation	[48]
Trimethylamine n-oxide	−0.94	−0.77	inducing M1 macrophage polarization	graft-versus-host disease	[49]
Phosphatidylcholine lyso alkyl 16:0	−0.98	−0.98	–	–	–

### 5.3 Effects of 3D clinostat on femur parameters in mice

**Fig. 6:**
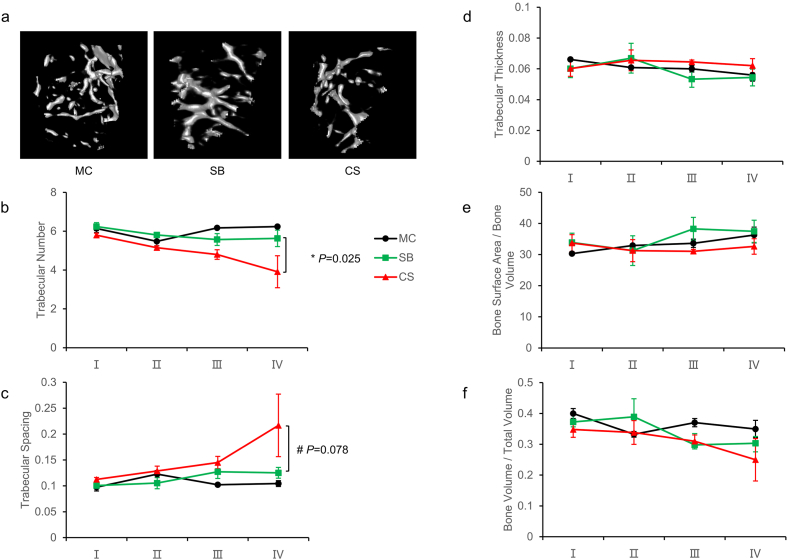
Effects of 3D clinostat on bone parameters in mice. (a) Representative micro-CT images at stage Ⅳ. (b) Trabecular numbers. (c) Trabecular spacing. (d) Trabecular thickness. (e) Bone surface area/bone volume. (f) Bone volume/total volume. Ⅰ: 1–3 weeks, Ⅱ: 4–6 weeks, Ⅲ: 7–9 weeks, Ⅳ: 10–12 weeks. *P < 0.05, #*P* < 0.1.

Femur parameters were analyzed, including bone surface area/bone volume, bone volume/total volume, trabecular number, trabecular spacing, and trabecular thickness. No significant differences in femur parameters between SB and MC mice were observed (Fig. 6a–f). It suggested when mice were stimulated with the survival boxes, time-dependent bone volume/total volume, trabecular thickness, trabecular spacing, trabecular number, and bone surface area/bone volume were unaffected. However, when mice were rotated in the 3D clinostat to simulate the effect of microgravity, there were clear differences between the SB and CS groups in femur Parameters. Compared with the SB group, there was a declining trend in time-dependent trabecular numbers (*P <* 0.05) while trabecular spacing showed an increasing trend (*P* = 0.078) in the CS group (Fig. 6b and c). These results indicated that simulating microgravity with a 3D clinostat led to bone loss. The longer the simulation period of microgravity with a 3D clinostat, the more severe the bone loss.

## 6 Discussion

Weightlessness is inevitable on a space mission and contributes to a series of damaging effects. Economics and technology have limited our ability to study the effects of weightlessness in space. Therefore, models that simulate the effects of microgravity (such as hindlimb unloading models) have become the preferred tools for studying weightlessness. However, these models also have some limitations [20]. For example, weightlessness occurs when the entire object floats completely in space, whereas hindlimb unloading model with the remaining forelimb loading only simulates partial weightlessness [20]. This method may not make the subject accept the overall weightlessness.

In our study, we used a 3D clinostat to simulate the effects of microgravity. On a clinostat, an organism is in a gravitational field and is subjected to a constant gravity vector, but because of the rotation of the clinostat, the direction of the gravity vector acting on the organism continuously changes, and the vector sum generated by one rotation (360°) is equal to zero, i.e., zero gravity [21,50]. The direction of the gravity vector changes rapidly so that the organism does not experience gravity, and the result is an effect similar to that of a microgravity environment [21,51].

To adapt to the microgravity, astronauts need to undergo a range of adaptive training before going into space, for example, parabolic aircraft flights and head down tilt. With the development of space industry, more and more experimental animals go into space. As one of the main purposes of the current studies is to focus on the effects of microgravity on physiological function, at present, there is no systematic microgravity adaptation training program for laboratory animals. However, for some special experiments and long-term experiments, it is necessary to train the animals properly to adapt to the microgravity environment to improve their conditions and survival rate. For this purpose, mice were trained to live on a 3D clinostat. Theoretically, it is possible to simulate microgravity on the ground by processing samples with 3D clinostat. It has been used on plants, cells, and *Caenorhabditis Elegans* [[22], [23], [24], [25], [26], [27], [28], [29]]. However, it was difficult for mice to keep immobile, considered as a mass point. Thus, we took advantage of the burrowing habits of the mouse and designed a survival box to keep the mice fixed on the 3D clinostat. Over a period of three months, the mice showed good tolerance without obvious injuries or abnormalities. It is suggested that using 3D clinostat to train mice is technically feasible.

Further, to explore the effects of simulated microgravity simulated by the 3D clinostat, we evaluated serum metabolomics and bone parameters. To exclude the effect of the SB itself alone, we analyzed the metabolomics of mice in the SB and MC groups, and the results showed no significant differences between the two groups (Fig. 2b). The results suggested that the SB would not interfere with our experimental results. For CS group mice, our results showed that mice treated with a 3D clinostat developed a stress response during the early stage Ⅰ, leading to a significant increase in the stage Ⅰ differential metabolites compared to other stages. Retrograde Endocannabinoid signaling is primarily affected. Studies showed [52] that CB1 receptor-endocannabinoid signaling was activated by stress and played a role in buffing or inhibiting the behavioral and endocrine effects of acute stress. Sachin Patel et al. found [53] that activation of CB1 cannabinoid receptors reduced anxiety-like behaviors in mice and further supported an anxiolytic role for endogenous cannabinoid signaling.

Subsequently, our results showed that KEGG signaling pathways activated in stage Ⅰ were recovered in the stage Ⅱ and Ⅲ, and the number of serum differential metabolites also decreased. It suggested that the mice gradually adapted to the 3D clinostat intervention in the stage Ⅱ and Ⅲ. Body weight results also supported this point. During long-term space operations [50], microgravity may affect intracranial physiological functions, such as intracranial pressure, spinal and neurocognitive performance, and brain edema induced by microgravity [54], resulting in neuro-ocular syndrome [51]. Irina Mikheeva et al. observed [55] that 30-day spaceflight had a significant effect on the structure of motoneurons of the trochlear nerve nucleus in mice. Xiao Wen Mao et al. found that the mice exposure to the spaceflight environment (Space Shuttle Atlantis, STS-135) could induce significant changes in protein expression related to neuronal structure and metabolic function [56]. The results from this study also showed that the nervous system was the most to be affected by long-term microgravity in the stage Ⅳ, that indicated by the activated KEGG pathways including Amphetamine addiction, Cocaine addiction, Dopaminergic synapse, Fatty acid biosynthesis, Melanogenesis and Prolactin signaling pathway. Consistent with it, long-lasting spaceflight (one month on the Russian Bion-M1 spacecraft) considerably affected the genetic control of the brain dopamine system in mice, while relatively short-lasting spaceflight (about 19 days on the biosatellites Cosmos 782 and 936) showed no significant changes in the dopamine level in rats [57,58]. The implication of the dopamine system in the regulation of movement, muscle tone and reward-related motivation suggests that the change of dopamine related signal pathways may contribute to the deleterious effect of spaceflight on skeletal muscle tone, locomotor activity and emotions that may alter in astronauts after long-term spaceflight. It is well known that mice as well as humans have vestibular organs in the inner ear. Movement of the clinostat would stimulate hair cells and lead to vestibular system disorder. Vestibular system has extensive connections with areas of the brain beyond the vestibulo-ocular and vestibulo-spinal reflexes, including motor control, multisensory integration, cognitive functions and emotional regulation [59]. It is reasonable to suppose that abnormal vestibular signals and sensory conflicts during rotation affected the function of the central nervous system. Adaptation of mice in 3D rotator may help mice adapt to vestibular perception abnormalities in space microgravity environment, just like the swivel chair training conducted by astronauts to prevent space motion sickness. Therefore, the mouse models built with 3D clinostat may provide an excellent model to explore the long-term effect of microgravity on nervous system. Besides, the results also suggest that it is very important to select appropriate training duration for 3D clinostat simulated microgravity, for example, 3 weeks for acute and stress effects, 4–9 weeks for short term effects without obvious stress reactions, and more than 10 weeks for long term and central nervous system related effects.

Our results also showed two overlapping metabolic pathways in the stage Ⅰ and Ⅳ: valine, leucine, and isoleucine degradation and 2-oxocarboxylic acid metabolism. Valine, leucine, and isoleucine [60], also known as branched-chain amino acids, usually act as nitrogen carriers to assist in synthesizing other amino acids required for muscle formation. Branched-chain amino acids have both synthetic and antide composition effects, which can help prevent protein decomposition and muscle loss [60,61]. Frederico Gerlinger-Romero et al. found [62] that Beta-hydroxy-beta-methylbutyrate (a leucine metabolite) can improve skeletal muscle function and protect bone from the harmful effects of fasting in Wister rats after fasting. 2-Oxocarboxylic acid metabolism [[63], [64], [65]] was closely related to osteoarthritis, myocardial infarction and bronchial asthma. Naiqiang Zhu et al. demonstrated [64] that 2-oxocarboxylic acid metabolism was highly correlated with osteoarthritis and can be used as a biomarker for early diagnosis of osteoarthritis. Valine, leucine, and isoleucine degradation and 2-oxocarboxylic acid metabolism were potential targets for using 3D clinostat to simulate microgravity effects, and change the dietary supply of branched-chain amino acids may help mice or astronauts adapt to microgravity environment.

Multiple studies showed that weightlessness causes bone loss in humans and mice. Bone parameters (e.g. bone trabeculae) were reduced, leading to an increased fracture risk [[66], [67], [68]]. To investigate whether 3D clinostat induced bone loss in mice, we compared bone parameters by CT imaging. Similarly, our results indicate that there are no significant differences in bone parameters between the SB and MC groups, ruling out the influence of the SB alone (Fig. 6). However, The CS group mice using microgravity simulated by the 3D clinostat clearly reduced trabecular number and increased trabecular spacing with time-dependent (Fig. 6). It suggested that 3D clinostat could cause bone loss by simulated microgravity. Bone loss is related to bone formation and bone resorption. Bone formation has been observed to decrease in most space flight studies, and bone resorption showed increase in humans in space and rodent models [[69], [70], [71], [72], [73], [74], [75], [76]]. Therefore, in our study, the decrease in trabecular number and increase in trabecular spacing may be due to the combined effect of decreased bone formation and increased bone resorption and it was time-dependent. Meanwhile, the bone effect seemed modest in the case of 3D clinostat, albeit small changes of trabecular number and trabecular spacing in the late stage. The modest bone loss is likely to be associated with the mechanical stimulation of the hind limb bones caused by the reflex grasping of the cage during the rotation process.

## 7 Conclusions

It was feasible to simulate the effect of microgravity on C57BL/6 J mice by 3D clinostat treatment. Bone loss (a reduction in the trabeculae number) agreed with previous studies. Additionally, serum metabolomes were changed with increasing 3D clinostat treatment time. The effects of microgravity simulated by a long time 3D clinostat are most affected in the nervous system. It may provide a new strategy in pre-launch training for mice to adapt to the microgravity environment in space and conducting relevant ground-based modeling experiments.

## Declarations

### Author contribution statement

Chenchen Song: Performed the experiments; Analyzed and interpreted the data; Wrote the paper.Taisheng Kang: Performed the experiments.

Kai Gao: Analyzed and interpreted the data; Contributed reagents, materials, analysis tools or data.Xudong Shi: Analyzed and interpreted the data.

Meng Zhang: Performed the experiments.

Lianlian Zhao: Analyzed and interpreted the data.

Li Zhou: Conceived and designed the experiments; Analyzed and interpreted the data; Wrote the paper.

Jianguo Guo: Conceived and designed the experiments; Contributed reagents, materials, analysis tools or data; Wrote the paper.

### Data availability

The data that supports the findings of this study are available from the corresponding author upon request.

## Funding

This research was supported by Aerospace Science and Technology Collaborative Innovation Center Project (BSAUEA5740600223), CAMS Innovation Fund for Medical Science (CIFMS, 2021-I2M-1–034), the National Natural Science Foundation of China (General Program, 82070103), the National Key R&D Program of China (2021YFF0703400).

## Ethical approval and consent to participate

Not applicable.

## Consent for publication

Not applicable.

## Declaration of competing interest

The authors declare that they have no known competing financial interests or personal relationships that could have appeared to influence the work reported in this paper.
